# Black heterosexual men’s resilience in times of HIV adversity: findings from the “weSpeak” study

**DOI:** 10.1186/s12889-023-15103-1

**Published:** 2023-01-27

**Authors:** Roger Antabe, Martin McIntosh, Erica Lawson, Winston Husbands, Josephine Pui-Hing Wong, Godwin Arku, Isaac Luginaah

**Affiliations:** 1 Department of Health and Society, University of Toronto Scarborough, 1265 Military Trail, M1C 1A4 Toronto, ON Canada; 2 Regional HIV/AIDS Connections (RHAC), 30-186 King Street, N6A 1C7 London, ON Canada; 3 Department of Gender, Sexuality, and Women’s Studies, Western University, 1151 Richmond Street, Lawson Hall Room, 3260, N6A 5B8 London, ON Canada; 4 Dalla Lana School of Public Health, University of Toronto, 155 College Street, M5T 3M7 Toronto, ON Canada; 5 Daphne Cockwell School of Nursing, Faculty of Community Services, Toronto Metropolitan University, Podium Building, Room POD-481, 350 Victoria St, M5B 2K3 Toronto, ON Canada; 6 Department of Geography and Environment, Social Science Centre, Western University, 1151 Richmond St, N6A 5C2 London, ON Canada

**Keywords:** African caribbean and black, Heterosexual, HIV, Resilience, Ontario, Canada

## Abstract

**Background:**

In Canada, heterosexual African, Caribbean and Black (ACB) men tend to suffer a disproportionate burden of HIV. Consequently, studies have examined the underlying contributors to this disparity through the nexus of behavioral and structural factors. While findings from these studies have been helpful, their use of deficit and risk models only furthers our knowledge of why ACB men are more vulnerable to HIV infection. Thus far, there is a dearth of knowledge on how heterosexual ACB men mobilize protective assets to promote their resilience against HIV infection.

**Methods:**

As part of a larger Ontario-based project called weSpeak, this study examined how ACB men acquire protective assets to build their resilience to reduce their HIV vulnerability. We analyzed three focus group discussions (n = 17) and 13 in-depth interviews conducted with ACB men using NVivo and a mixed inductive-deductive thematic analyses approach.

**Results:**

The findings show that ACB men mostly relied on personal coping strategies, including sexual abstinence, to build resilience against HIV. Interpersonal resources such as family, friends, and religious communities also played an important role in constructing ACB men’s resilience. ACB men bemoaned their lack of access to essential institutional resources, such as health services, that are important in managing HIV adversity.

**Conclusion:**

Based on these findings, there is an urgent need for HIV policy stakeholders, including service providers, to engage the ACB community in the design of intervention programs. Additionally, addressing the socioeconomic disadvantages faced by ACB communities will increase the capacity of ACB men to develop resilience against HIV.

## Background

The low prevalence rate (0.2%) of human immunodeficiency virus (HIV) in Canada tends to mask the realities of HIV risk in the country [1]. Some studies have suggested that sexual minorities and racialized groups report similar HIV prevalence rates as the so-called HIV-endemic areas of low-income countries [2]. People of African, Caribbean, and Black (ACB) descent in Canada are particularly vulnerable to HIV exposure. For instance, while people of ACB descent represent less than 5% of Canada’s population, they made up more than 25% of new HIV cases in 2019 [3]. Furthermore, their HIV vulnerability is often framed as ‘self-inflicted’, thus foreclosing opportunities to engage them through health interventions [4, 5].

Studies have pointed to numerous challenges the ACB community in Ontario faces in accessing health care, including disconnection from healthcare spaces. For ACB people living with HIV, this may have implications for accessing HIV counseling and treatment services that are vital to reducing their viral loads to Undetectable = Untransmittable (U = U) [6, 7]. The poor health experienced by the ACB community has also raised human rights concerns, given the suggestion that it is underpinned by race-based discrimination. For instance, experiences of economic discrimination and underrepresentation in medical professions and service delivery, and biases against racialized groups in health decision-making, contribute to ACB people’s poor health [8]. In addition, race-based discrimination in employment, education and housing have been identified as critical determinants of health for Black Canadians [9].

Among heterosexual ACB men, studies that have attempted to understand their HIV vulnerability have done so by examining the contributing role of their social and sexual behaviours. Such studies often tend to focus on Black men’s practices of traditional masculinity which is observed to deter some men from seeking health services and support [10–12]. By prioritizing behavioral risk factors as the main contributors to their HIV vulnerability, assets that are critical for building HIV resilience among ACB men, including those living with HIV, are overlooked. In addition, the structural and systemic conditions that may be contributing to their HIV vulnerability are not explored. In this paper, ‘resilience against HIV’ refers to activities and practices of ACB men that help to minimise their exposure to HIV. For those living with HIV, resilience relates to practices that help to manage HIV as a lifelong chronic condition. In line with evidence suggesting that people’s resilience against HIV may reflect the quality of their structural environment, some scholars [13–16] have called for a re-examination of ACB men as active agents who, with appropriate support and resources, can address their disproportionate burden of HIV. Indeed, earlier studies in Ontario, Canada, have stated that ACB men are aware of their HIV vulnerability and therefore, were proactive in meeting their own health needs and making the necessary adjustments and choices to reduce their exposure to the virus [5, 17]. Despite this evidence, studies continue to use deficit and risk models that only explain the disproportionate burden of HIV among Black men (and women). Thus far, no study uses a resilience and asset-based approach to examine how HIV seronegative ACB men reduce their exposure to HIV, and how those living with HIV cope with and overcome the stress associated with their diagnosis.

This study forms part of an Ontario-based research project called ‘weSpeak’ which engaged stakeholders in critical dialogue through innovative and community-based approaches to address HIV vulnerability in the ACB community in Ontario. Using the settings of London, Ontario, this paper examines the sources and acquisition of protective assets by heterosexual ACB men in building resilience against HIV, and for ACB men living with HIV to live with HIV as a lifelong chronic condition. We adapted our study to the theoretical construct of ‘critical resilience’ published by Traynor [18]. Critical resilience is discussed as encompassing Black men’s awareness of their own sociopolitical circumstances in a racially structured society. Based on this awareness and building on our previous studies on Black men’s resilience [19], we see resilience in the ways they mobilize themselves as individuals and as a community in resistance to conditions that compromise their health and wellbeing [19]. Thus, Black men’s resilience is constructed as being rooted in the quality of their socio-environmental conditions, such that their capacity to reduce their exposure to HIV and/or manage HIV as a lifelong chronic condition is further shaped by these prevailing conditions and resources.

## ACB men and resilience against HIV

Resilience is differently defined, but generally connotes the ability of an individual or a community to bounce back from adversity [20]. It may also be defined as constituting the protective assets at the disposal of individuals, that cushion and equip them with the capacity to recover from adversity [20, 21]. In its earliest application, resilience was linked to people’s innate qualities including self-efficacy, hardiness, social competence, and self-control, which made them ‘naturally’ adapt to temporary setbacks [22, 23]. However, broader conceptualization of resilience also includes its intersection with socio-environmental factors, and any supportive resources that can help individuals to gain protective assets to be able to thrive through adversity [24, 25].

An individual’s ability to reduce their exposure to HIV may be based on their accumulated protective assets, which is dependant on their access to, and the quality of, resources in their sociocultural environment. Studies suggest that Black people’s higher prevalence of HIV may be partly due to a lack of awareness and low uptake of HIV testing and treatment [26, 27]. Emphasising the role of information as a contributor to HIV resilience, the Ontario Advisory Committee on HIV/AIDS [28] posits that declining HIV diagnoses among highly exposed groups, including men who have sex with men (MSM) and persons who inject drugs (IDU), is attributable to policies that intensified information dissemination amongst them. This is because unhindered access to relevant culturally-grounded information equips individuals with the requisite knowledge to reduce their exposure [29, 30]. Similar arguments are made for the frequent use of HIV-related health services, where those with easy access to appropriate facilities are better positioned to accumulate protective assets to build resilience. Frequent users of HIV preventive health services are exposed to information on HIV transmission based on which they make informed decisions on reducing their exposure. People who are living with HIV may initiate counselling and treatment services that contribute to increase their resilience against the everyday challenges of living with the virus [31]. In Canada, though ACB men may be vulnerable to HIV, they are among the least visible group in AIDS Service Organizations (ASO) and health care spaces. This could be linked to their inability to access factual information on HIV transmission, thus adversely impacting their accumulation of protective assets to build resilience against HIV [32].

Overcoming adversity also involves having a strong network of family, friends and interpersonal resources within existing social and cultural structures that buffer individuals’ resilience [20, 33–35]. The presence of social and cultural support systems that prioritize HIV testing, treatment, and access to information may encourage individuals in these support systems to increase their resilience against HIV [36]. ACB people’s sociocultural and social network characteristics are sometimes invoked to explain why they may be more vulnerable to HIV. For instance, HIV stigma, poor interaction with health care spaces, homophobia as well as the endorsement of traditional masculinities, are theorized to prevent heterosexual ACB men from accumulating enough protective assets against HIV [27, 37]. It is further suggested that ACB men do not readily discuss their sexual and health vulnerabilities which may foreclose their interaction with useful resources to accumulate protective assets to fight HIV [38, 39].

Unfortunately, most discussions about ACB men’s HIV risk are bereft of structural contexts despite their overarching influence over people’s ability to acquire protective assets in the face of HIV adversity [22, 33]. Structural factors as used in this study refer to the social and health policy decisions at the level of government that directly and indirectly shape the health outcomes of individuals [40, 41]. Furthermore, the term ‘vulnerability’ as used in our study is indicative of the ways health policies and the social conditions of heterosexual ACB men increase their risk of poor health [42]. Highlighting the role of structural factors in the burden of HIV among ACB men, Husbands et al. [16] argued that ACB men are aware of the structural conditions that contribute to their poor health, but they have not been engaged by health policy makers. Particularly, ACB men are excluded from health care policies that should provide them with health services to support their acquisition of assets for building resilience to HIV. Regarding these challenges, what we do not fully understand is how heterosexual ACB men are responding to their HIV vulnerabilities beyond the common behavioral connotations. Very few studies have interrogated the ACB community’s health practices, and how they may be mobilizing protective assets for the purpose of building resilience against HIV. This study, therefore, examined resilience trajectories and practices of ACB men in London Ontario, with the aim of contributing to health policy literature on resilience to HIV. In particular, the findings will improve our understanding of the protective assets characteristics and mobilization dynamics among ACB communities and their potential for designing HIV-related health policy.

## Study context

London, Ontario, has a population of 383,822 according to the 2016 population census of Canada. It is as one of the fastest growing cities in the country, ranking as the 6th and 15th largest city in Ontario and Canada, respectively. As of 2016, people of ACB descent constituted 2.6% of the city’s total population, which is higher than similar sized cities in Canada [43, 44].

Over the last decade, London has had one of the highest prevalence rates of infectious diseases, such as hepatitis C and HIV, in Ontario. Evidence from both the Public Health Agency of Canada and the London-Middlesex Health Unit—the local body responsible for administering and managing health care in the city—places residents of London at heightened risk of infection. HIV infection rates in London increased from 5.9 cases per 100,000 in 2005 to 9 per 100,000 in 2015 [45]. Similar trends were recorded for hepatitis C infections in the city. These disturbing trends of infection led the London-Middlesex Health Unit to declare a ‘medical emergency’ in 2016 as part of the strategies to reduce new infections in the city. The main strategy was to increase the supply of clean needles and create safer spaces for supervised and safe drug injection for persons that injected drugs. Missing from this initiative, however, is a strategy for addressing HIV transmission including infection by heterosexual contact that represents majority of HIV diagnoses in the ACB community [4]. Despite heterosexual ACB men constituting one of the key HIV vulnerability groups, there is no specific policy to reduce new infections among them.

Although access to primary health care in London has improved over the years, a 2016 report suggested some sub-populations in the city experienced barriers in accessing healthcare services [46]. Ethnocultural and racial minority populations including ACB people continue to face challenges in the access and utilization of healthcare services. These challenges are likely made worse by the depressed socioeconomic status of ACB men [46]. In an earlier study on health equity and access in London, some members of the ACB community emphasized the challenges they face in the city’s health care system which compel them to resort to seeking healthcare services outside the city [47]. To understand the HIV needs of ACB populations in London, Baidoobonso and colleagues [48] called for a more structurally focused approach where HIV risk, vulnerability and resilience can be situated in the structural determinants of health.

## Methods

This study forms part of the qualitative phase of weSpeak research project implemented in four Ontario cities—London, Windsor, Ottawa and Toronto—between 2015 and 2020, to understand the pathways to better include heterosexual ACB men in community-based responses to HIV. The present study focuses on how heterosexual ACB men in London who are HIV seropositive and negative position themselves and mobilise protective assets to overcome HIV adversity. In addressing this research objective, we examined ACB men’s health affirming activities and practices in the face of HIV adversity. We also sought to identify existing strategies and protective assets or resources, and to understand how they mobilized these resources to build resilience against HIV. Participants also identified experiences that weakened their ability to build resilience against HIV. The methods for this study were approved by the Western University Non-Medical Research Ethics Board (NMREB) in 2015. All methods for this study were carried out in accordance with relevant guidelines and regulations of Western University’s NMREB.

### Participant recruitment

Participant recruitment for focus groups (FG) and in-depth interviews (IDI) took place between April 2016 to March 2017. Informed by our interpretation of resilience as ‘critical resilience’ [18] we sought to understand the varied resilience experiences, trajectories and practices of heterosexual ACB men in London, Ontario. With this, we aimed to unpack narratives of heterosexual ACB men in relation to their heightened risk to poor health outcomes including HIV. Furthermore, we sought to explore how heterosexual ACB men as a social group act to reduce their increased vulnerability to HIV adversity, and how they navigate their social environment as a means of resisting the conditions that predispose them to poor health. Thus, recruitment was carefully designed and rolled out to interview participants from varying backgrounds, including diverse socioeconomic, geopolitical, ethno-cultural, and immigration status. Those with both positive and negative HIV serostatus were also included. To qualify to participate in the research, the prospective participants self-identified as heterosexual ACB men, were aged 16 or older, spoke English or French, and lived in London. Given that the targeted participants are considered a ‘hard to reach’ population, the study employed a five-pronged approach in the recruitment. First, the study identified ACB men’s common spaces including Black barbering salons, churches, mosques, and African-Caribbean restaurants where project notices and flyers were distributed. Those interested in the research made initial contact with the research coordinator for recruitment in the study. Second, the leadership of ethno-racial groups including those from sub-Saharan Africa (SSA) and the Caribbean were contacted and informed about the purpose of the research. The leaders then informed their membership about the research and invited them to contact the research coordinator for additional information and to be screened for their eligibility to participate in the study. Third, notices for prospective participants to contact the researchers were posted in the spaces of health service providers including the Regional HIV/AIDS Connection (RHAC) and some selected hospitals in London. Fourth, recruitment also took place during community activities and events that brought members of the ACB community together. For instance, researchers were present at activities marking Black History Month and a health fair organized for racial minorities by the RHAC. Research assistants shared information about the research with ACB men they encountered in public spaces. Interested individuals contacted the research coordinator for initial screening to determine their eligibility to participate. We obtained informed consent from all participants who met the eligibility criteria. Demographic details of participants are provided in Table 1.

**Table 1:** Socio-demographic characteristics of in-depth interviews and focus group participants

Participants Characteristics	Frequency (n = 30)	Percentage %
**Ethno-identity**		
African	12	40.0
Caribbean	10	33.3
Black	8	26.7
**Region of Birth**		
Africa	7	23.3
Caribbean	5	16.7
Canada	16	53.3
Other	2	6.7
**Immigration Status**		
Canadian Citizen	23	76.7
Landed immigrant/Permanent citizen	4	13.3
Refugee/Protected Person Claimant	2	6.7
Other/Student Study Visa	1	3.3
**Age**		
16–24	8	26.7
25–38	12	40.0
39+	10	33.3
**Employment Status**		
Employed	12	40.0
Self-employed	6	20.0
Student/part time	7	23.3
On disability	3	10.0
Unemployed	2	6.7
**Annual Income (CAD$)**		
<20,000	18	60.0
≥ 20,000	12	40.0
**Educational Attainment**		
High school or less	12	40.0
Some college/university/completed college/university	18	60.0
Masters or higher		
**Marital Status**		
Married or common law	10	33.3
Single (never married/divorced/separated)	20	66.7
**HIV Status**		
HIV+	3	10.0
Never tested/don’t know/HIV-	27	90.0
**Religion**		
African Traditional	1	3.3
Christian	22	73.3
Muslim	3	10.0
Other/no religion	4	13.3

### In-depth interviews and focus groups

Heterosexual ACB men interested in the study were invited to participate in either a focus group discussion (FG) or an in-depth interview (IDI). For FGs, participants were organized into three groups based on their age to ensure resonance among group members [49, 50]. Overall, three (3) focus groups were conducted involving individuals in age categories 16–24 (n = 5), 25–38 (n = 5), and 39 and older (n = 7). Thirteen (13) in-depth interviews with individuals aged between 16 and 67 were conducted, to delve further into the nuances emerging from themes in the three FGs. IDIs were conducted with ACB men living with HIV to prevent the accidental disclosure of their HIV status in the setting of a group discussion. Generally, the IDI was useful in providing depth and contextualizing findings from FGs. It was also useful to use this medium to discuss views that may not be suitable to a group setting. Overall, FGs lasted between 60 and 90 minutes while IDIs lasted between 45 and 60 minutes. Sessions for both FGs and IDIs began with individuals giving both verbal and written consent to be audio-recorded. Participants were assured their identities would be protected and anonymized in research papers and reports. To achieve this, names of participants and their demographic data were collected on separate forms. Each participant was then assigned a unique alphanumeric code for the interviews, and this helped to anonymize their personal information on transcripts. Both interview recordings and transcripts were kept on a flash drive. The flash drive and the forms containing participants’ demographic information are kept in a safe and secure locker with only the principal investigator having access to these materials. In addition, only pseudonyms were used with quotes in reporting the research findings. Participants were made aware of their right to have the recorder switched-off at any point in the interview, to refuse to answer any question, and to withdraw from the study at any point. FGs were held at the Regional HIV/AIDS Connections (RHAC) while IDIs took place at spaces identified by participants as safe and appropriate for the interview. All FGs and IDIs were conducted in English.

Guided by a checklist of questions, participants in both FGs and IDIs discussed issues related to how they acquire protective assets to build HIV resilience. They first discussed their understanding of resilience and how the concept informs their efforts against HIV. Specifically, they linked the concept of resilience to their everyday lived experiences in the City of London, Ontario. They identified existing resources for coping with HIV, including individual protective assets and those at the interpersonal level. They further discussed the role of institutional resources in building personal and community resilience to HIV. The core questions that guided the discussions included the following: (1) how are ACB men accessing information and health services related to HIV?; (2) what specific activities or programs target ACB men in an attempt to keep them informed about available community resources; (3) what resources are available for those who test seropositive to HIV?; and (4) what challenges do ACB men face, and how do these challenges impede their ability to acquire protective assets to build resilience to HIV? An honorarium of $20 was given to each participant to cover the cost of transportation to the interview venues. Interview guides for the weSpeak study were published in Bryce [51] in 2018.

### Data analysis

All audio recordings from the interview sessions were transcribed verbatim by research assistants under the guidance of the research coordinator. The transcripts were then exported into NVivo, a qualitative software for analysis. Guided by the overall project objective and mandate of weSpeak, our approach to data analyses was informed by a transformative paradigm as we seek to draw social and political attention to the health needs of heterosexual ACB men in Canada [52]. With this approach, centering the needs of marginalized and structurally disadvantaged populations is central to the research as it invokes social change in communities through research and engagement with key stakeholders [53]. We employed mixed inductive-deductive thematic coding where the process of theme identification was informed by both resilience theoretical constructs and the data itself [7, 54, 55]. Team members read the transcripts to familiarize themselves before proceeding to code and identify themes related to our research objectives. To ensure consistency in coding among three team members, coded transcripts were exchanged between team members to crosscheck and verify that the coded discussions were representative of their assigned themes and were consistent with other transcripts. Some of the research co-PIs with extensive experience in qualitative research were given copies of the coded transcripts for their independent review.

## Results

The analyses revealed several themes on how ACB men cope and thrive through HIV adversity. These themes were tied to participants’ conceptualization and understanding of resilience and their acquisition of protective assets to build resilience against HIV. We found that heterosexual ACB men used both internal (or personal) and external resources to build resilience against HIV. While internal resources were expressed in personal characteristics such as openness to discuss one’s vulnerability and willingness to connect to others in the ACB community, external ones were primarily defined by interactions with family members and friends.

Findings also revealed that the key sources of protective assets to building resilience against HIV were differently perceived and prioritized based on participants’ personal experiences and sociodemographic characteristics. For instance, in both FGs and IDIs, younger ACB men emphasized internal assets, including self-efficacy, hardiness, social competence, and self-control, to be foundational to building resilience. Still, younger ACB men recognized the importance of external and institutional support in developing individual and collective/community resilience against HIV. In contrast, older ACB men, as well as those with spouses and dependants, emphasized family and social support from ACB community members as a critical source of external assets in building resilience against HIV. Overall, ACB men identified lack of institutional support in helping them to accumulate protective assets in building resilience against HIV. It was also revealed that, while ACB men living with HIV shared similar sources of resilience with other interviewees,  they named interactions with ASOs as additional sources of protective assets to coping with HIV. However, they recognised that these interactions were infrequent and could be improved to meet their needs.

Several structural dynamics foreclose the community’s ability to accumulate protective assets for building resilience against HIV. These were mostly tied to the lack of governmental and institutional support in positioning the ACB community to access quality healthcare resources. Specifically, race-based discrimination in employment as well as the non-prioritization of the health needs of ACB people by state institutions and health policymakers, worked together to reduce their capacity to accumulate protective assets against HIV. Regardless of these structural dynamics, we found that religion was an important platform employed in mobilising both internal and external resources against HIV. Religious groups such as churches provided a sense of community, and an important medium for ACB men to discuss their vulnerabilities and acquire protective assets. Heterosexual ACB men draw on their everyday experiences to propose ways in which their resilience against HIV can be improved. The next section further elaborates these key findings. Findings are supported with direct quotes from study participants to provide context for further clarity. At the end of each quote, the participant’s age and type of interview (FG or IDI) are provided.

### Participant’s conception of resilience

Participants explained that HIV-related resilience generally includes internal resources or personal protective assets which position them to freely discuss and share their feelings of vulnerability with family members and acquaintances. However, there was a recognition that internal resources or personal protective assets of resilience have to be formed. This suggests the importance of external resources in ACB men’s social environment for adequately positioning them to respond to their HIV vulnerability. As one participant stated.

We have innate determination, it is given, we have that. But it needs to be formed into resilience with respect to adverse circumstances and adverse environments. And with diseases like HIV/AIDS, our resilience has to be formed, so we channel our resilience into positive ways to get through something like HIV [FG, 25–38 years group].

Experiences of adversity in the past provided participants with the ability to identify support systems in overcoming the impact of HIV. For some ACB men therefore, resilience to HIV meant access to HIV information and testing.

You can build resilience, you know, get tested, learn about HIV, know your status, those types of things. It protects yourself. We [ACB men] should be getting ourselves tested as a priority.” [IDI, 35 years old].

Participants also conceptualized resilience to encompass protective behaviours such as condom use during sexual intercourse. Akwasi, a 45-year-old Caribbean man, summarised what he perceived to constitute the most important component of resilience against HIV for ACB men: *“Somebody don’t have sex because they don’t want to have children, but the condom is the umbrella of resilience for black men.”*

ACB men perceived that being informed about the realities of their HIV vulnerability is foundational to acquiring protective assets to build resilience against HIV. Accepting the realities of their vulnerability could work to compel them to identify resources that can be used to acquire protective assets against HIV.

Acceptance is the fact that as an African Canadian Black man, I am in fact vulnerable to HIV. As well, if I don’t utilize [HIV] information, I become more vulnerable” [FG, 25–38 years group].

Access to information for this participant is a key protective asset and a resource that is foundational to equipping members of this community with the capacity to build resilience against HIV infection.

### Individual intrinsic resilience and coping mechanisms

Heterosexual ACB men employed several individual intrinsic strategies and initiatives to reduce their exposure to HIV. These included sexual abstinence as highlighted in the following quote:

I feel right now in this very moment, I am 100% not vulnerable to HIV because of choices such as sexual abstinence [IDI, 25 years old].

A 32-year-old year old recent immigrant to London affirmed abstinence as a resilience tool *“…ever since I got to Canada, I have not had sex with anybody [for fear of getting infected with HIV]. And that’s almost a year now. I don’t rush things. I always want to be sure what I do.”* This indicates a capacity for thoughtful self-reflection which may include a role for abstinence. For those who may not be abstaining from sex, limiting sexual activities to one partner, preferably in stable relationships worked to reduce their vulnerability and build their resilience against HIV.

Most of the sex I have had comes in an actual relationship. And you think that doesn’t really seem like it means much in reducing risk of HIV infection, but one, it reduces the number of partners you have; and two, it usually puts space in between your partners [IDI, 40 years old].

It was also revealed that issues relating to trust and HIV stigma are discouraging some heterosexual ACB men from freely discussing their HIV vulnerabilities. They thereby resort to dealing with feelings of vulnerability by participating in or initiating activities that engages them.

When I’m feeling vulnerable, I just like to listen to music. I know a lot of people might want to speak to someone about it, but again with a lot of the stigmas around HIV, a lot of people are afraid to talk to someone about it. I used to play sports a lot to help myself deal with those type of things when I’m stressed [IDI, 25 years old].

Those without such strong intrinsic capacity for self-control and to engage broader aspects of their personality or inclinations may find themselves in challenging circumstances if their environments also lack the external resources to deal or cope with such vulnerabilities. For instance, those who found it difficult to discuss their vulnerability with others may suppress and overcome adversity by using illicit substances, which could increase their vulnerability to HIV.

### Resilience through community

Emerging from the findings was the revelation that the larger ACB community plays an essential role in helping ACB men to acquire protective assets to build resilience against HIV by providing them support and a sense of community. In addition, building social networks within the ACB community worked to keep members informed. This may include information on where to get help both within and outside the ACB community.

The thing I realized being a Canadian and growing up in this society is that it depends on who you know to succeed. If you are not accessing different social networks out there, then you are not in the know, and if you are not in the know how can you benefit or grow as a person?…. I have got this great big social network because I allowed myself to be a little vulnerable which was very hard for me to do [FG, 39 + years group].

While some ACB men may shy away from building networks in their community, it is considered a necessary resource in the settings of London which has a small population of Black people. This was captured in the remarks of a 57-year-old participant in a FG.

When it comes to your community, you don’t think about interacting with them, you have to put in effort. It is the same with drawing support of the Black community, you have to try because you are not just going to go to the grocery store and see 20 black people, and 5 of them you know.

This recognition by ACB men acknowledges a sense of common vulnerability and the need to mobilize protective resources through their networks of ACB people. Improving connection and networking in the ACB community as a protective asset against adversity was echoed by another participant in an IDI.

I find it is beneficial to our brothers and sisters, to say hey, you know what, I’m going to get vulnerable [i.e., stressed, and exposed] why don’t you share your struggles with me, I will share mine with you. It’s like give a little to get a little [IDI, 25 years old].

Another participant also stated the benefits of keeping such social networks. Beyond one’s immediate family.

That’s where it starts, actually exposing or revealing that side of you that you are vulnerable to somebody who you trust. …You can probably tell a pastor or have a conversation with a near friend who has values and get his perspective and his advice. You find some way to get help and don’t just say, OK, I don’t have any help” [FG, 39 + years group].

ACB men did not only conceptualize community to include friends, family, and resources within the reach of their immediate environment, but it also involved virtual and online communities. Through these virtual communities, they share uplifting messages, discuss community relevant information, and provide social support and opportunities among members.

For some participants with transnational ties, communicating with contacts in origin countries was also important in overcoming HIV adversity. This was noted by a participant as follows:

I often bring up some issues…personal stuff like my health and things like this [HIV vulnerability], there isn’t anything that my sisters don’t know. I also have my cousin’s husband who is in Jamaica, I talk with. I have other friends outside Canada I speak with. So, I have a fair network of people [FG, 39 + years group].

This quote underscores the important role of transnational ties and communication on the health and wellbeing of immigrants in their destination areas in Canada.

### Spirituality and religion as sources of resilience

Participants identified religious beliefs as a critical resource in their resilience trajectories. Many suggested that for ACB men who were going through difficult times, religious teachings and spirituality helped them to overcome adversity. Churches, mosques, and religious groups are able to offer emotional support to members in overcoming challenges such as substance dependency that could heighten vulnerability to HIV. By so doing, religious groups serve as important platforms in building resilience against HIV.

I got baptized and dedicated my life to the Lord. And it was always a struggle. It’s like forget about all my old life [drug addiction and sex with multiple partners], this is my new life, this is who I am, this is my identity, this is what I have been waiting for…it [has] been 8 months ago I gave up everything of my old life. [IDI, 25 years old]

For a city like London which has a relatively small degree of racial diversity, Black churches are crucial in bringing the ACB community together to fight adversity or provide support to members in times of adversity. This is observed in a quote below.

When I look at this, I still see that the church is a sort of pillar of the community… that sort of props the community up, it must be the starting point. I go back to churches because they seem to be the pillar. I mean everybody seems to get there [FG, 39 + years group].

The contribution of religious institutions to ACB men’s resilience against HIV was also through the messages preached to congregants. For instance, some ACB men believed that those who heed to religious teachings about sexual relationships tend to reduce their exposure to HIV. A participant commented in an IDI:

From a spiritual perspective, I said I was Christian. I guess if someone is in a situation where they are very spiritual, sex outside committed relationships is probably not something that is encouraged. But if someone was very spiritual, I suppose they would be discouraged from having indiscriminate sex to begin with [IDI, 25 years old].

Most participants agreed that while religious teachings may not directly touch on HIV, the application of these messages to their everyday lives helps adherents to protect themselves and build resilience against HIV. A 57-year-old participant in an in-depth interview remarked *“Well, if you are a religious person then depending on your religion you shouldn’t be practicing polygamy [i.e., implied as multiple sexual partners]. So, you should be only dedicating yourself to one woman, not cheating on her and that will decrease any possibilities of [getting] HIV.”* Preaching empathy and sexual abstinence, and the importance of healthy living may encourage individuals to protect themselves from HIV.

### Resilience for ACB men living with HIV

Participants living with HIV used health and institutional services to accumulate protective assets during their resilience trajectories. For instance, a participant living with HIV recounted how with the help of some agencies, he accepted the realities of his HIV serostatus and initiated treatment.

…I was distant from a lot of people; it was something that I never wanted to get myself into. And I went to this location on a retreat, and I never forget it, a men’s retreat, and it really opened my eyes [IDI, 45 years old].

ACB men receiving these services may over time develop trust and establish friendship with service providers, which helps them in overcoming stress and depression associated with living with HIV. In more extreme situations, individuals can access mental health services from health providers. Another seropositive participant recounted his experience in an interview:

Because of the way I was infected, there is a psychological component to it. It can bring out mental issues to the surface. And I think that for the longest time before I was infected, I was an angry individual. And I was an angry individual probably for about 8 years, until I went and got help for post-traumatic stress disorder [through the agency] [IDI, 32 years old].

For this participant, referral services helped him to adjust and to overcome some of the challenges of living with HIV. Through this, the participant was able to come to terms with his HIV status and seek treatment. The revelation that HIV treatment allowed seropositive ACB men to have healthy lives makes their interactions with ASOs crucial to the process of building resilience against HIV. A participant living with HIV commented during an interview:

I look at it this way, a kind of benefit from having HIV is that our health is monitored, it’s incredibly monitored. So, when you’re going and you’re doing your blood work every three months, you can find out or know of the possible things coming down the tube [IDI, 45 years old].

While services offered by ASOs emerged as important for ACB men living with HIV, it appeared that those who were not living with HIV, or were unaware of their serostatus, were not connected to these services. In most cases, they did not know the services these organizations provided or where they were located.

### Strategies to improve ACB men’s resilience

Participants proposed ways the ACB community could be assisted to acquire protective assets to fight HIV adversity. These proposed measures mainly focused on improving their connection to ASOs which they considered valuable to accessing information and other useful resources, such as testing, that can build their capacity to adequately respond to HIV. This is emphasized by a recent ACB immigrant:

I was talking about understanding the realities of the virus. I think a lot of people [i.e., ACB men], including myself have no knowledge around the ways in which you can contract the virus and ways in which once contracted, you could treat the virus [IDI, 25 years old].

Connecting ACB men to ASOs had the potential to not only inform them about HIV transmission, but also encourage HIV testing and treatment for those living with the virus. It became apparent from the interviews that testing also has an added benefit of promoting collective resilience in ACB communities, as observed by a 40 year old in an IDI: *“I think you have to hit bottom, rock bottom to realize who is your friend, who is around, what can you do so when you are right back to the top you know how to deal with it.”* To ensure increased testing among ACB men, participants suggested the use of innovative ways of disseminating information about HIV to the community. For instance, the use of virtual platforms like social media and other mediums such as churches, mosques, community leaderships and entertainment figures could improve HIV information and knowledge in the community. A participant in a FG commented:

People are so influenced by the media. Everyone’s searching their phones these days…let’s have HIV information here, let’s have awareness, information there, and let’s pump it out like that [FG, 25–38 years group].

Another participant emphasized the strategy of increasing HIV information dissemination using prominent entertainment figures in the community. In a FG, a 25-year-old participant had this to say: *“Because I do videos myself, I find that when there is a famous person in a video people watch. For example, if Eminem is talking about HIV and the vulnerabilities, boom, you got like 10 million people following instantly”*. This may also require the political mobilization of Black leaders and the community to put pressure on the state to be more proactive in meeting the health needs of the ACB community.

Furthermore, structural barriers appeared prominent in explaining the difficulties ACB men face in mobilizing protective resources to improve their resilience against HIV. Participants stated that reducing the social and economic vulnerabilities of ACB men can help build resilience, as noted below:

On a broader scale, it is important to lessen the stratification of society. Basically, improve social economic standards for the poor in society which by and large are made up of Blacks and the black community, and then, we can actually go to school and learn instead of just having information thrown at us when we can’t concentrate because stuff at home is just messed up [FG, 25–38 years group].

The need for improved access to social services for the ACB community as a pathway to acquiring protective assets was echoed by a 45-year-old immigrant. *“We need access to decent educational facilities, better schools, better home environments and less incarceration of our people”.* Additionally, he acknowledged the need for community mobilization to dialogue with the state and community stakeholders on providing resources that can help build resilience against HIV. A participant in a FG noted:

We need more Black leaders; we need people to really get out there and go hard. Because if you don’t see a thousand black faces who are looking at you like what are you going to do for us then you are not going to do anything. You are going to be like yeah guess what, there are 15,000 black people in London, and you only see 15 in a given day if you really look hard. Then you don’t care about black people. [IDI, 35 years old]

From the perspectives of ACB men, strategies that take a comprehensive approach to building resilience against HIV must consider ACB people’s socioeconomic vulnerabilities based on the social structuring and inequitable justice system in Canada. Community mobilization to demand more from stakeholders will also ensure that ACB men are sufficiently resourced to become resilient against HIV.

## Discussion

In this study, we examined the sources of protective assets used by heterosexual ACB men in building resilience against HIV. The findings suggest that ACB men face several and intersecting structural barriers that adversely impact their access to improved health, testing and treatment services and information. Regardless of this structural disadvantage, ACB men still demonstrate resilience through the acquisition of protective assets in their daily lives and interactions with family, friends, and community members. The resilience trajectories and practices of ACB men center on their internal assets and community social support, with little to no involvement of state institutions and resources. However, for ACB men living with HIV, some form of institutional support and connection with service providers emerged as critical in coping with or building resilience against HIV.

In demonstrating critical resilience [18, 19], heterosexual ACB men in London, Ontario, acknowledge the circumstances that underpin their vulnerability to HIV. They respond to these as individuals and as a community by organizing their activities and interactions to increase their capacity to be resilient against HIV. Through this, ACB men challenge and resist skewed narratives about their capacity and fortitude to be resilient. Furthermore, ACB men show that despite governmental and institutional neglect over their health needs, they can still mobilize and respond to adversity as a marginalized group. In our study setting, heterosexual ACB men defined resilience to cover personal protective assets, family, friends, and social networks, and they employed a multiplicity of strategies to cope with and manage HIV. While institutional and health resources were recognized by ACB men as crucial protective assets that can empower them to build resilience against HIV, it emerged that only ACB men living with HIV may be in touch with these institutional resources. Thus, although ACB men defined resilience to incorporate connection to ASOs, these organizations were not targeting them, and this affected their capacity to build resilience against HIV. Importantly, local religious communities emerged as important facilitators in ACB men’s acquisition of protective assets in their resilience against HIV.

ACB men’s conceptualization of resilience is consistent with Wood et al.‘s [25] view that resilience is a multidimensional concept that transcends individual intrapersonal qualities to embrace the quality of resources within their social environment in responding to adversity. Access to accurate information on HIV, designed and implemented with other strategies that ensure Black men are able to achieve their full health potential, can be useful in helping to build ACB people’s capacity to be resilient against HIV [56, 57].

Employing individual strategies such as abstinence from sexual intercourse and limiting sexual activities to one partner in a stable relationship, was another major pathway for participants to build resilience against HIV. Importantly, some of these practices were linked to spirituality and religiosity that also emphasized sexual abstinence and discouraged partner concurrency. While monogamy and abstinence linked to spirituality and religiosity has been found to help reduce HIV vulnerability among individuals [58], other studies such as Isler et al. [36] have opined that HIV risk as constructed and preached by religious beliefs, expectations and doctrines may not be a realistic strategy for reducing HIV vulnerability among people. This may be emanating from the observation that religious backed messages and construction of HIV risk are often pegged as a moral call rather than an emphasis on activities such as condom use that are useful for reducing HIV vulnerability [59, 60]. In this regard, despite attempts by religious denominations to be part of efforts to reduce ACB men’s exposure to HIV, persons perceived not to be practicing abstinence or living with HIV may face judgement and moral condemnation which works to deplete their capacity to be resilient against HIV. As suggested by Bryant-Davis et al. [61] and Bradley et al. [62], given how integral Black religious denominations are to ACB communities, their messaging on HIV should promote non-judgemental and non-stigmatizing understanding of HIV vulnerability. Thus, introducing stigma reduction messages as part of religious messages will be useful in promoting resilience among ACB men in their respective communities. Furthermore, churches, mosques and religious organizations can provide teaching and social support options that enable ACB men to choose a value-guided lifestyle that positively influence their sexual decision-making [63]. This strategy may be more effective in promoting ACB men’s resilience against HIV as it will encourage access to accurate and inclusive information on safer sex, HIV prevention, counselling, and treatment. Overall, the use of sexual abstinence as a strategy to reduce exposure to HIV among study participants demonstrates their resolve to be resilient against HIV.

Religious communities like churches also served as a reliable source of interpersonal assets for the resilience trajectories of ACB men. Through teachings and emphasis on spirituality, ACB people living with HIV, as well as those going through difficult times, were given a glimpse of hope. This has been found to be effective in developing positive adaptive mechanisms to overcome HIV adversity specifically in Brazil and Belgium [65, 66]. In North America, Black churches have mobilized the ACB community to fight adversity including Jim Crow, and they remain important spaces for resisting structural oppression, racism and discrimination [67, 68]. Although some studies implicate Black churches for their complacency in contributing to high HIV prevalence in their communities, they remain important pillars and rallying points for ACB men in drawing strength to fight HIV. More recently, Black churches may have taken up the challenge of becoming more proactive about HIV as they organize voluntary counselling and testing services, and provide emotional and psychological support to members [36, 69].

These research findings offer an opportunity for health policymakers and ASOs to build collaborations with the leadership of religious institutions and use these religious platforms to engage with membership [69]. This could happen in the following ways. First, there is the need to collaborate with the leadership of local religious groups/organizations to understand the HIV health needs of their congregants. This can serve as the basis for thinking and developing collaborative strategies to engage Black communities and ACB men. Second, ASOs and health promoters can work with religious institutions and groups to complement HIV-related activities such as testing and counseling sessions that are occasionally organized for congregants. This could provide the medium to interact with and educate community members on HIV and where to access important services that are crucial for building resilience against HIV. Third, given the evidence that leaders in local religious organizations hold some influence over congregants through their sermons and interactions, ASOs could develop short to long-term training programs on HIV that target religious leaders. This could increase their knowledge base about HIV transmission and treatment, and address issues of HIV stigma that are reinforced in the sermons of some of these leaders.

Participants identified personal networks as instrumental to helping them acknowledge and overcome adversity [70, 71]. However, Diprose [20] suggested that prioritizing individuals’ and communities’ internal qualities to overcome adversity follows a neoliberal ideology where affected individuals and communities are expected to get by with little to no involvement of the state. This neoliberal understanding of resilience may particularly impact marginalized populations (including ACB men) that are inequitably resourced to withstand adversity. There is a need to increase their access to important social resources that would further improve their capacity to overcome HIV adversity.

In London, Ontario, where ACB people constitute less than 3% of the city’s population, building social networks with other ACB people facilitated access to support from community in times of adversity. Though there is an argument for intrapersonal assets [35], ACB men in London also used links to the ACB community to navigate protective asset-building in the city. Their networking includes transnational ties and virtual communities where they drew strength to respond to HIV. Given evidence that heterosexual ACB men may be disconnected from state resources that could aid them in acquiring protective assets against HIV [13], it is not surprising that non-institutionalized resilience strategies (e.g. sexual abstinence) are used to overcome and cope with HIV vulnerability. Among persons living with HIV, virtual communities have emerged as an important platform for promoting adaptive approaches to HIV [see 59, 60]. These findings align with Drushel’s [72] suggestion that marginalized groups use these channels to circumvent inaccessible traditional and formal sources of HIV information and support. ACB men’s use of virtual platforms in their resilience trajectories has positive implications for reaching them remotely with interventions and strategies during challenging and difficult circumstances including outbreaks and pandemics such as COVID-19.

To promote and help strengthen Black men’s resilience to HIV, ASOs and HIV-related service outlets may engage with the ACB community through innovative strategies. This would require the involvement of community and religious leadership and other role models to make outreach campaigns more effective in the community [35, 36, 73]. Importantly, ACB men in the study underscored the urgency of addressing structural barriers and discrimination that limit their access to socioeconomic and health resources which directly deplete their protective assets to overcome HIV in the community. Thus, improved economic and educational opportunities in ACB communities can translate into increased HIV awareness, more frequent visits to preventive health care spaces, and increased utilization of services at ASOs [5, 74]. Such improvement will directly build ACB men’s HIV resilience and empower them with protective assets to adequately respond to HIV.

### Study limitations

Our study has some noteworthy limitations. First, given that our study used a convenience sampling framework, the perspectives of the participants captured in the findings may be missing some of the nuances of the experiences of ACB men who could not be recruited through this approach. Furthermore, despite attempts to reduce the power dynamics inherent in focus groups, it is possible that the sensitive nature of some of the issues being discussed may have prevented some participants from sharing their experiences. Finally, the analysis did not take into consideration the role of migration origins in the resilience trajectories of ACB men as earlier studies have posited that these categories impact the lived realities of ACB men in North America, including Canada.

## Conclusion

Heterosexual ACB men’s ability to mobilize protective assets to build resilience against HIV has seldom been of interest to researchers and policy makers. Our analysis presents an important opportunity for policy makers to potentially expand some of these resilience building practices into HIV interventions targeted at the ACB community. For instance, access to relevant information from ASOs emerged as a key protective asset for ACB men, especially those living with HIV. Collaborating with Black community members could work to increase the presence and interaction of ASOs with community members. Through this strategy, ASOs can increase their presence in barbershops, Black grocery shops, and cafes to disseminate HIV information and engage with community members on workable ways to increase their resilience against HIV. In addition, recruiting community members to lead HIV information sessions at community events would be a useful way to reach more people with HIV information. Furthermore, the revelation that ACB men who are not living with HIV or are not aware of their HIV serostatus mostly rely on personal and intrapersonal protective assets in building resilience against HIV points to the urgent need to target and engage them to overcome HIV in the community. Beyond HIV testing and information dissemination, a holistic approach to improving ACB men’s health outcomes must be considered. There is accumulated evidence that racism and race-based discrimination undermine health and wellbeing among Black Canadians [9,75]. It is, therefore, necessary to address the problem of social structuring that exacerbates ACB men’s vulnerability to HIV as it restricts their access to economic and health resources. Taken together, our findings establish that ACB men are investing in resilience against HIV, and with improved collaboration with ASOs and other health policy stakeholders, they are poised to overcome their individual and community HIV adversity.

## Data Availability

The datasets generated and analysed during the current study are not publicly available due to restrictions placed on it by the Western University Non-Medical Research Ethics Board but are available from the corresponding author on reasonable request.

## Abbreviations

ACB: African, Caribbean and Black
HIV: Human Immunodeficiency Virus
AIDS: Acquired Immunodeficiency Syndrome
ASO: AIDS Services Organizations
RHAC: Regional HIV/AIDS Connections
IDI: In-depth Interview
FG: Focus Groups
